# Theoretical Investigation of the Fusion Process of Mono-Cages to Tri-Cages with CH_4_/C_2_H_6_ Guest Molecules in sI Hydrates

**DOI:** 10.3390/molecules26237071

**Published:** 2021-11-23

**Authors:** Shuxian Wei, Siyuan Liu, Shoufu Cao, Sainan Zhou, Yong Chen, Zhaojie Wang, Xiaoqing Lu

**Affiliations:** 1 School of Science, China University of Petroleum, Qingdao 266580, China; wshx@upc.edu.cn; 2 School of Materials Science and Engineering, China University of Petroleum, Qingdao 266580, China; b20140005@s.upc.edu.cn (S.C.); zhousn@s.upc.edu.cn (S.Z.); wangzhaojie@upc.edu.cn (Z.W.); 3 School of Geosciences, China University of Petroleum, Qingdao 266580, China

**Keywords:** hydrate, density functional theory, multi-cage fusion, mixed guest molecules

## Abstract

Owing to a stable and porous cage structure, natural gas hydrates can store abundant methane and serve as a potentially natural gas resource. However, the microscopic mechanism of how hydrate crystalline grows has not been fully explored, especially for the structure containing different guest molecules. Hence, we adopt density functional theory (DFT) to investigate the fusion process of structure I hydrates with CH_4_/C_2_H_6_ guest molecules from mono-cages to triple-cages. We find that the volume of guest molecules affects the stabilities of large (5^12^6^2^, L) and small (5^12^, s) cages, which are prone to capture C_2_H_6_ and CH_4_, respectively. Mixed double cages (small cage and large cage) with the mixed guest molecules have the highest stability and fusion energy. The triangular triple cages exhibit superior stability because of the three shared faces, and the triangular mixed triple cages (large-small-large) structure with the mixed guest molecules shows the highest stability and fusion energy in the triple-cage fusion process. These results can provide theoretical insights into the growth mechanism of hydrates with other mono/mixed guest molecules for further development and application of these substances.

## 1. Introduction

Natural gas hydrates are non-stoichiometric compounds constructed by water and gas molecules. Due to the wide distribution in permafrost layers and beneath seafloor, this substance has the potential to become an alternative source to fulfill the growing need for nature gas in the global market and solve the energy crisis in the near future [1,2,3]. The most common type of hydrates is structure I (sI), which concludes two small cages (owing to 12 pentagonal faces, denoted by 5^12^, s) and six large cages (owing to 12 pentagonal faces and 2 hexagonal, denoted by 5^12^6^2^, L) per unit cell formed at a low temperature and high pressure [4,5]. These cages are formed by the hydrogen bond between water molecules and the interaction between host (water) and guest (such as CH_4_, CO_2_, H_2_, N_2_, C_2_H_6_, C_3_H_8_, and so on) molecules [6,7,8,9,10,11,12,13,14]. The large number of holes in the structure is conducive to the storage of guest molecules, such as hydrogen and carbon dioxide [15,16,17], which provides an effective pathway for exhaust gas capture. To better utilize natural gas hydrates (as energy sources and gas storage material) it is essential to understand the microscopic mechanism of how hydrate crystalline grows in the existence of guest molecules. 

It has been revealed that the sI structures growing up gradually from mono-cages to triple cages (tri-cages) is a common process, due to the intermolecular interactions of the cages [9,18,19]. Mono-cages, including small cages (5^12^) and large cages (5^12^6^2^), play an important role for building double cages and multi-cages. Double cages own three combinations based on two main kinds of mono-cages [20]. Water cages are likely to share more faces during the nucleation of CH_4_ hydrates, meaning that triangular tri-cages are more stable than linear tri-cages [19], yet few examples have been reported for the influence on hydrates growth with other mono/mixed guest molecules. Generally, the guest molecules play an important role for supporting host water cages and avoiding structural collapse. Different guest molecules have different influences on the stability of hydrate structures, as well as the fusion behavior of multi-cages. Studies have been promoted to explore the influence of mixed guest molecules in hydrates [8,16,21,22,23,24]. Su et al. reported that structure II (sII) type clathrate crystal is thermodynamically stable when the hydrates are partially or fully occupied with three different guest molecules (CH_4_, C_2_H_6_, and C_3_H_8_) [7]. Furthermore, experimental data shows that sI will be formed by the existence of C_2_H_6_ while sII can only be discovered when the concentration of C_2_H_6_ lies in between 2% and 22% [21], which indicates that C_2_H_6_ is essential for the formation of sI hydrates. However, this lacks micro-mechanism study on the stability of sI hydrates with C_2_H_6_ and mixed CH_4_/C_2_H_6_ as guest molecules during the nucleation stage. Hence, it is necessary to explore the stabilization and fusion trend of hydrates with CH_4_/C_2_H_6_ guest molecules.

In this study, pure CH_4_, pure C_2_H_6_, and mixed CH_4_/C_2_H_6_ were selected as guest molecules to explore the fusion process from mono-cages to tri-cages in sI hydrates. We find out that the large cage (5^12^6^2^) is prone to capturing larger volume C_2_H_6_ guest molecule based on its suitable pore spaces. Large (5^12^6^2^) and small (5^12^) cages are likely to contain C_2_H_6_ and CH_4_ molecules in the double-cage fusion process, respectively. On the basis of stability energy and fusion energy of double cages, the nucleation process of the double cage is formed by 5^12^ and 5^12^6^2^. The triangular triple-cage structure may be the main form of tri-cages with CH_4_/C_2_H_6_, due to the three sharing faces. The mixed triple cages (tri-LsL) composited by two large cages and one small cage have higher stabilization and fusion energy during the triple-cage fusion process. According to the calculations of related thermodynamic energy, it is obvious that the introduction of an extra 5^12^6^2^ cage help the tri-cage formation based on stable and mixed double cages. The low thermodynamic energy corresponds to stable structure, rendering to search formation mechanism. Consequently, the fusion process from mono-cages to triple cages, following an order of 5^12^6^2^ (L), mixed double cages (Double-Ls), and mixed triple cages (tri-LsL), is thermodynamically favored. The formation micro-mechanism of hydrates with mixed CH_4_/C_2_H_6_ guest molecules is investigated as well, which could provide theoretical guidance for actual hydrate mining.

## 2. Models and Methods

All calculations were carried out by density functional theory (DFT) with the Gaussian 09 program [25]. The B3LYP functional [26] with D3 correction (Becke–Johnson damping) [27] was adopted for its regularity and dispersion corrections. For the main group elements (C, H, O), the all-electron 6-31+g(d,p) basis sets [28] was applied to describe the system electronic structure. To simulate the real hydrate formation process, temperature and pressure were set at 273.15 K and 30 atm, respectively. The convergence criteria of maximum force and maximum displacement were set to be 4.5 × 10^−4^ and 1.8 × 10^−3^ bohr in structure optimization, and corresponding root mean square were 3.0 × 10^−4^ and 1.2 × 10^−3^ bohr.

In order to describe the thermodynamic stabilities of hydrates, the stabilization energy (E_sta_) is applied in this study [19], and the E_sta_ per H_2_O molecule (E_(sta-p)_) is used to compare the relative stabilities of different structures [19,29], which is given by

(1) $$E_{\mathrm{sta}}{=\mathrm{mE}}_{\mathrm{guest}}{+\mathrm{nE}}_{H_{2}O}{\;-\;E}_{\mathrm{hydrate}}$$

(2) $$E_{\mathrm{sta}-p}{=E}_{\mathrm{sta}\;}/\;n$$

where m, n represent the number of guest molecules and water molecules in hydrate cages, respectively. E_guest_, $E_{H_{2}O}$, and E_hydrate_ are the thermodynamic energy of the single guest molecule, water molecule, and hydrate. The interaction energy (E_int_) can reflect the binding strength between guest molecules and water cage structures, which is defined as [30]

(3) $$E_{\mathrm{int}}{=(\mathrm{mE}}_{\mathrm{guest}}{+E}_{\mathrm{water}\;\mathrm{cage}}{)\;-\;E}_{\mathrm{hydrate}}$$

where E_water cage_ represents the energy of water cages without guest molecules. On the other hand, the ability of water cages capturing guest molecules can also incarnate the crystal growth of hydrates. Therefore, the capture energy (E_c_) and the capture energy per guest molecules (E_cp_) can be expressed as [9]

(4) $$E_{c}{=E}_{\mathrm{hydrate}}{\;-\;\mathrm{mE}}_{\mathrm{guest}}{\;-\;E}_{\mathrm{water}\;\mathrm{cage}}$$

(5) $$E_{\mathrm{cp}}{=(E}_{\mathrm{hydrate}}{\;-\;\mathrm{mE}}_{\mathrm{guest}\;}{-\;E}_{\mathrm{water}\;\mathrm{cage}})/m$$

To estimate the stabilities of multi-cage structures in the fusion process, the cage fusion energy (E_fusion_) was first proposed by Khan [29,31], and the calculation formula of fusion energy is as follows

(6) $$E_{\mathrm{fusion}\;}{=E}_{\mathrm{sta}(\mathrm{multi}-\mathrm{cages})}\;{-\;(E}_{\mathrm{sta}\left(\mathrm{cage}\;1\right)}{+E}_{\mathrm{sta}\left(\mathrm{cage}\;2\right)}\;\;-\;\mathrm{shared}\;\mathrm{ring}\;\mathrm{size}\;\times \;\;E_{{\mathrm{sta}-p(\mathrm{cage}\;1\;\mathrm{or}\;\mathrm{cage}\;2,\;\mathrm{whose}\;E}_{\mathrm{sta}-p}\;\mathrm{is}\;\mathrm{lower})})$$

where E_sta(multi-cages)_, E_sta(cage 1)_ and E_sta(cage 2)_ represent the stabilization energy of multi-cages (double cage or tri-cage), the two parts are divided by multi-cages, respectively. The size of the shared ring is equal to the number of water molecules on the shared face of cage 1 and cage 2. Thus, the more positive E_fusion_ value implies higher stability of multi-cages in the fusion process. On the basis of double-cage fusion, triple-cage fusion was treated as a fusion process of the double cage and mono-cage, which will be further discussed in the results and discussion.

## 3. Discussion

### 3.1. The Influence of CH_4_/C_2_H_6_ Guest Molecules on Single Cage

**Figure 1:**
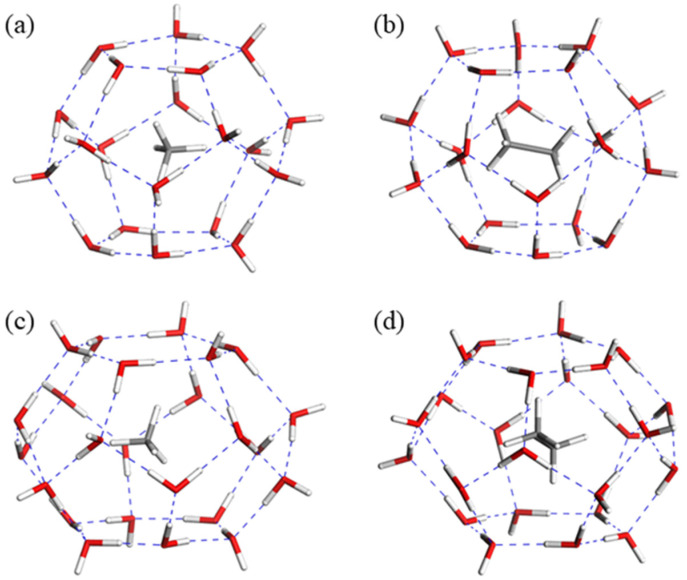
The structural configuration of small (5^12^) cages with (**a**) methane and (**b**) ethane, and large (5^12^6^2^) cages with (**c**) methane and (**d**) ethane.

**Figure 2:**
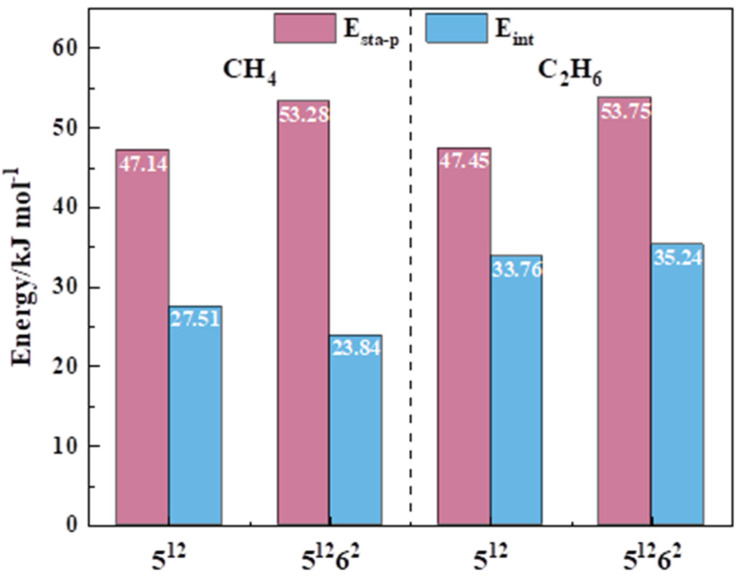
The stabilization energy per H_2_O molecule (E_sta-p_, kJ mol^−1^) and interaction energy (E_int_, kJ mol^−1^) of CH_4_ and C_2_H_6_ guest molecules in small (5^12^) and large (5^12^6^2^) cages.

The research of a basic single-cage structure is crucial for probing the influence of different guest molecules on sI hydrate. The formation mechanism of the single cage has been revealed as a ring-expansion process for the small cages and a layer-separation mechanism for the large ones [32]. As shown in Figures S1 and S2, the formation of 5^12^ and 5^12^6^2^ with CH_4_ guest molecules is simulated based on the above mechanism. The structural configurations of small and large cages with CH_4_ and C_2_H_6_ are displayed in Figure 1, in which the guest molecules occupy the center of dodecahedron and tetrakaidekahedral water cage structures after optimization. To obtain a better picture, the parameters representing the thermodynamic stability of single cages are summarized in Figure 2. Compared with other calculations for a mono-cage with CH_4_ guest molecules by different methods, it provides evidence to support the accuracy of this work (Table 1). Detailed information of the H_2_O–guest molecule equilibrium distances during the fusion process of mono-cages to tri-cages with different guest molecules can be obtained from Supplementary Materials Table S1.

As can be seen from Figure 2, when the guest molecule is CH_4_, the E_sta-p_ of 5^12^6^2^ is around 6 kJ mol-1 higher than that of 5^12^. When it comes to E_int_, an opposite situation (E_int_–5^12^ > E_int_–5^12^6^2^) takes place. The results are in good consistence with the previous report [32], which indicates that the small cage is more feasible in the early stage of nucleation because of larger E_int_, but the large cage would be the decisive factor for the formation of sI methane hydrate crystals owing to its higher E_sta_. As for hydrate with C_2_H_6_, the large cage is more favored than small cage, referring to both structural and energy factors. The E_sta-p_ of 5^12^6^2^ with C_2_H_6_ guest molecule is 53.75 kJ mol^−1^, which is slightly higher than that of 5^12^ (47.45 kJ mol^−1^). The 5^12^6^2^ with a E_int_ of 35.24 kJ mol^−1^ exhibited a superior interaction between the guest molecule and water cage than the 5^12^ cage (33.76 kJ mol^−1^). This is most likely due to the large molecular volume of C_2_H_6_, which reduces the distance and enhances the interaction between the guest molecule and water cage. On account of these results, a large cage containing a C_2_H_6_ guest molecule would play a significant role in the nucleation and growth process of sI hydrate. Hence, regardless of the influence of different guest molecules on the E_int_, a large cage is critical for forming the sI hydrate crystal structure. 

### 3.2. The Stabilities of Double Cages with CH_4_/C_2_H_6_ Guest Molecules

**Figure 3:**
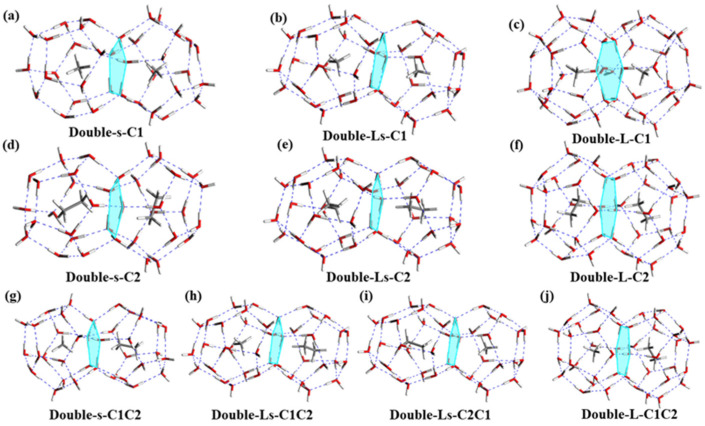
The structural configuration of (**a**,**d**,**g**) Double-s, (**b**,**e**,**h**,**i**) Double-Ls, and (**c**,**f**,**j**) Double-L, shared with pentagon, pentagon, and hexagon water rings, respectively.

In the growth process of a double-cage hydrate, the cage unit will possibly occur one by one [18]. In addition, the multi-cage fusion plays a significant role during the formation of sI hydrate [19]. For double cages, there are three combination types involved in the small-cage (5^12^) and large-cage (5^12^6^2^) fusion process: (1) double-small-cage fusion; (2) double-large-cage fusion; (3) mixed-small- and large-cage fusion, which are denoted as Double-s, Double-L, and Double-Ls, respectively. It has been confirmed that if the number of shared rings is greater, the double cages will be more stable [19,20]. As shown in Figure 3, both Double-s and Double-Ls feature the same character of two cages sharing one pentagon ring while the two cages of Double-L share a hexagon water ring instead. It is also observed that two mono-cages of one double cage interact with each other by hydrogen bonds in the face-sharing water ring.

As can be seen from Table 2, the E_sta-p_ of the double-cage hydrates with CH_4_ or C_2_H_6_ follow the order of Double-L > Double-Ls > Double-s. Combining with the E_sta-p_ of mono-cages, there is a new sequence for structural stability: Double-L > Double-Ls > 5^12^6^2^ > Double-s > 5^12^, which indicates an advantageous trend for the growth of sI hydrate. What’s more, further evidence shown in Table S2 indicates better stability of the double cage with two guest molecules than with the single guest molecule, which further proves the above conclusion. Moreover, the C_2_H_6_ guest molecule has advantages in stabilizing hydrates. Since two different guest molecules are placed in the double cage, Double-Ls can be divided into two types: Double-Ls-C1C2 (Ls-C1C2) and Double-Ls-C2C1 (Ls-C2C1), in which the large cage contains CH_4_, small cage contains C_2_H_6_ for Ls-C1C2, large cage contains C_2_H_6_, and small cage contains CH_4_ for Ls-C2C1, respectively (Figure 3). It shows that Ls-C2C1 have higher E_sta_ than Ls-C1C2 (55.21 kJ mol^−1^ vs. 54.89 kJ mol^−1^, Table 2), which indicates that water cages with guest molecules of suitable volume own better stability.

Besides E_sta_, capture energy (E_c_) is an important parameter for judging hydrate structural stability. The E_c_ of double cages in Table S2 describe the ability of empty and half-full double cages to capture a single guest molecule. Owning to the distance between host and guest molecules, Double-s is ready to capture CH_4_ while Double-L focuses on C_2_H_6_ (Table 2). As for the CH_4_ guest molecule, empty and half-full Double-s cages exhibit superior performance with E_c_ of −26.98 kJ mol^−1^ and −28.43 kJ mol^−1^ (Table S2). Double-L with C_2_H_6_ and mixed CH_4_/C_2_H_6_ as guest molecules have better performance comparing to other Double-L structures. Similarly, the large cage of Double-Ls is prone to capture C_2_H_6_ and the small cage captures CH_4_. Therefore, the guest molecules contained in the hydrates are closely related to the pore size of the water cage.

### 3.3. The Fusion of Double Cages with CH_4_/C_2_H_6_ Guest Molecules

The fusion energy (E_fusion_) produced by two single cages fusing into one double cage is summarized in Table 2. When the guest molecule consists of a single component (CH_4_ or C_2_H_6_), the Double-Ls structure displays a thermodynamic advantage for fusion than the other three double-cage structures. In particular, small and large mono-cages containing CH_4_ are most likely to fuse together with an E_fusion_ value of 155.81 kJ mol^−1^.

Considering the influence of mixed guest molecules, a molecular ratio of CH_4_:C_2_H_6_ = 1:1 is applied to analyze the fusion behavior of the hydrate double cage. Similar to the double cage with a single guest molecule, Double-L with mixed CH_4_/C_2_H_6_ exhibits better stability than the other double-cage structures. As for Double-Ls, the large cage is prone to hold the C_2_H_6_ molecule, which can be proved by the higher E_sta-p_ of Ls-C2C1 than Ls-C1C2. Moreover, empty double cages all display a priority for capturing C_2_H_6_ in the mixed guest gas (Table S2). The double-cage fusion process with mixed guest molecules have a similar trend with a single component in E_fusion_, following an order of Double-Ls > Double-s > Double-L. For Double-Ls cages, Ls-C2C1 exhibits higher E_fusion_ (155.87 kJ mol^−1^) than that of Ls-C1C2 (150.27 kJ mol^−1^), indicating that it is more favorable for a large cage to capture C_2_H_6_ and a small cage to capture CH_4_. Compared with single guest component, the Double-s structure with mixed guest molecules CH_4_/C_2_H_6_ shows poor performance in the fusion process, while the Double-L structure exhibits slight superiority. As for the mixed double cage with mixed guest molecules, Double-Ls-C2C1 displays higher potential than others in the double-cage fusion process. The results imply that the mixed double cages exhibit stronger trend than others in the case of fusion. Moreover, Ls-C2C1 has a similar value of E_fusion_, with Double-Ls containing CH_4_ as the only guest molecule, which indicates the C_2_H_6_ guest molecule has the same potential as a methane hydrate in the double-cage fusion process.

### 3.4. The Stabilities of Triple Cages with CH_4_/C_2_H_6_ Guest Molecules

The formation of sI hydrate follows a continuous fusion process of multi-cages. As Double-Ls have higher stability, the third cage reserves two possibilities in forming a triple cage: small-large-small (tri-sLs) and large-small-large (tri-LsL) triple-cage structures. Different from linear tri-cages with two sharing faces, triangular tri-cages possess more stable structural features with three sharing faces [19]. In this work, we analyzed the influence of three different combinations of guest molecules (CH_4_, C_2_H_6_, and CH_4_/C_2_H_6_) on the stability of mixed tri-cages. 

**Figure 4:**
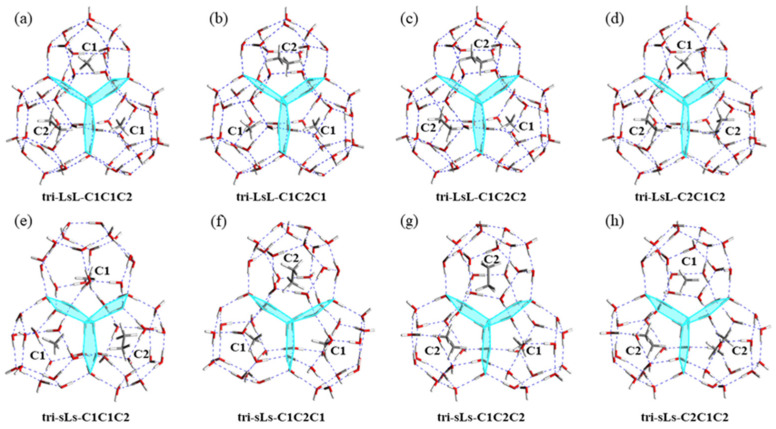
The optimized tri-cage structures, in which pentagonal water rings were shared in two small cages and mixed double cages, and hexagonal water rings were shared in two large cages. (**a**–**d**) Tri-cage structures consisting of two large cages and one small cage. (**e**–**h**) Tri-cage struc-tures consisting of two small cages and one large cage.

The optimized configuration of tri-cage structures (tri-sLs-C1 represents two small cages and one large cage with CH_4_ as guest molecule) are shown in Figure S3. A triangular tri-cage structure is formed with every two cages having one shared surface. There are three shared pentagonal faces in tri-sLs with one large and two small cages, while a hexagonal shared face appears in tri-LsL, due to neighboring large cages. When the guest molecule is just CH_4_ or C_2_H_6_, tri-LsL exhibits superior thermodynamic stability to tri-sLs (Table 3). Moreover, the larger volume of C_2_H_6_ enhances the interaction between the water cages and the guest molecules, which increases the stability of hydrate cages. There are eight combination patterns for the two tri-cage structures with mixed guest molecules, and the optimized configurations can be seen from Figure 4. As shown in Table 3, the values of E_sta-p_ for tri-sLs with mixed guest molecules are in the range of 54.51 kJ mol^−1^ to 54.80 kJ mol^−1^, which is lower than that of the tri-LsL structures (56.76–57.36 kJ mol^−1^). As for the tri-LsL structures, tri-LsL-C1C2C1 and tri-LsL-C2C1C2 exhibit the worst and the best performance in stability, respectively, which indicates that the small cage is prone to capturing CH_4_ and the large cage prefers to contain C_2_H_6_.

### 3.5. The Fusion of Double Cages to Triple Cages with CH_4_/C_2_H_6_ Guest Molecules

Based on the superior E_fusion_ of mixed double-cage fusion, the fusion of tri-cages occurs between the mono-cage and mixed double cage. There are two different triangular tri-cages merging differently with the third cage. When the third cage is a small one, it provides two pentagonal shared faces to form tri-sLs structures. When the third cage is a large one, it provides a pentagonal and a hexagonal shared face to form tri-LsL structures. Considering the more hydrogen bond interaction of the hexagonal shared face, tri-LsL structures can exhibit higher fusion trend than tri-sLs with CH_4_ or C_2_H_6_ guest molecules, which is consistent with the data summarized in Table 3. The result indicates that C_2_H_6_ helps the tri-cage structures to fuse easily as a single component guest molecule.

For tri-cages with mixed guest molecules, the E_fusion_ values are somewhere in between the structure with single CH_4_ and C_2_H_6_ as guest molecules. But this trend is better reflected in tri-sLs rather than tri-LsL. C_2_H_6_ plays an important role as the guest molecule in the fusion process for tri-sLs structures. It is obvious that tri-LsL structures with mixed guest molecules are more competitive in fusion than that of tri-sLs structures in the case of higher E_fusion_. Thus, the composition of the cage structures is also an important role in the fusion process of tri-cage structures. Furthermore, tri-LsL-C2C1C2 has the highest E_fusion_ (245.37 kJ mol^−1^) with CH_4_ in the small cage and C_2_H_6_ in large cages, which indicates that the mono-cage-holding guest molecule with a suitable size is the key factor for multi-cage fusion. The interaction between guest molecules and mono-cages plays a critical role in the process of multi-cage fusion. Hence, the triangular tri-cages with guest molecules fitting into cages with proper sizes have the highest stabilization and fusion energy. As a result, our theoretical study could provide a possible mechanism analysis for the fusion of mono-cages to tri-cages with CH_4_/C_2_H_6_.

## 4. Conclusions

In gas hydrates, the guest molecules play an important role in supporting the host water cages. There are complex gas components in the environment where gas hydrates are formed. It is of great significance for exploring hydrate formation mechanisms to analyze the influence of different guest molecules on the fusion process, from mono-cages to tri-cages. In this work, we select CH_4_ and C_2_H_6_ and their combination as the guest molecules, in order to analyze the stability of mono-cages and multi-cages and the fusion trend from mono-cages to tri-cages. We get the following conclusions:

(1) Small cages have advantages in structure, while energy is the advantage for large cages. According to these results, large cages play significant roles in the second-step formation of sI hydrate. On the basis of larger volumes of C_2_H_6_, the interaction between guest molecules and water cages are further improved. The large cage containing C_2_H_6_ is the most stable of the mono-cage structures.

(2) As for double cages, the large cage has the advantage in structural stability. Double-L with C_2_H_6_ makes full use of the interaction between the large cage structure and C_2_H_6_, which exhibit the optimal stabilization energy. Double-Ls with mixed guest molecules (Ls-C2C1) has the best performance in multi-cage fusion. 

(3) For the fusion process of double cages to tri-cages, tri-LsL-C2C1C2 exhibits superior properties both in stabilization and fusion energy. This is in full compliance with the rules, that is, appropriate holes of water cages can hold suitable volume of guest molecules. The tri-cage structures with two large cages and one small cage can adapt to different conditions to achieve structural stability in complex mixed guest molecules.

Our theoretical calculation results describe the fusion process of mono-cages to tri-cages with the different guest molecules and analyze the impact of different guest molecules on the stability and fusion trend of hydrate cages. This study provides a theoretical basis for exploring the influence of different guest molecules on the stability of hydrates in practical applications. 

## Figures and Tables

**Table 1:** The equilibrium distances in the stable geometries of the mono-cages with a CH_4_ guest molecule, obtained by different methods.

Method	Basis Set	d_O-C_ (Å)	Reference
MP2	Aug-cc-PVTZ	3.508	[33]
PBE-TS	TNP	3.473	
B3LYP	6-311++g(2d,2p)	3.671	
B97-D	6-311++g(2d,2p)	3.491	
B3LYP	6-31+g(d,p)	3.684	This work

**Table 2:** The stabilization energy (E_sta_, kJ mol^−1^), stabilization energy per H_2_O molecule (E_sta-p_, kJ mol^−1^), capture energy (E_c_, kJ mol^−1^), capture energy per guest molecule (E_cp_, kJ mol^−1^), and fusion energy (E_fusion_, kJ mol^−1^) of three guest molecules (CH_4_, C_2_H_6_, and mixed CH_4_/C_2_H_6_) in the double-cage structure.

Guest Molecule	Structure	E_sta_	E_sta-p_	E_c_	E_cp_	E_fusion_
CH_4_	Double-s	1780.12	50.86	−55.41	−27.71	130.00
	Double-Ls	2141.74	54.92	−51.08	−25.54	155.81
	Double-L	2339.38	55.70	−49.64	−24.82	101.58
C_2_H_6_	Double-s	1789.15	51.12	−64.45	−32.23	128.27
	Double-Ls	2152.63	55.20	−61.97	−30.99	150.77
	Double-L	2364.07	56.29	−74.32	−37.16	106.47
CH_4_/C_2_H_6_	Double-s	1783.62	50.96	−58.92	−29.46	127.35
	Ls-C1C2	2140.82	54.89	−50.16	−25.08	150.27
	Ls-C2C1	2153.12	55.21	−62.46	−31.23	155.87
	Double-L	2351.56	55.99	−61.81	−30.91	102.45

**Table 3:** The stabilization energy (E_sta_, kJ mol^−1^), stabilization energy per H_2_O molecule (E_sta-p_, kJ mol^−1^), and fusion energy (E_fusion_, kJ mol^−1^) of three guest molecules (CH_4_, C_2_H_6_, and mixed CH_4_/C_2_H_6_) in tri-cage structures.

Guest Molecule	Structures		E_sta_	E_sta-p_	E_fusion_
CH_4_	tri-LsL	C1C1C1	3070.29	56.86	235.90
	tri-sLs	C1C1C1	2776.97	54.45	163.77
C_2_H_6_	tri-LsL	C2C2C2	3092.03	57.26	240.62
	tri-sLs	C2C2C2	2795.97	54.82	168.80
CH_4_/C_2_H_6_	tri-LsL	C1C1C2	3083.75	57.11	243.23
		C1C2C1	3065.12	56.76	231.65
		C1C2C2	3078.14	57.00	238.55
		C2C1C2	3097.27	57.36	245.37
	tri-sLs	C1C1C2	2779.89	54.51	163.61
		C1C2C1	2791.20	54.73	166.62
		C1C2C2	2794.62	54.80	166.96
		C2C1C2	2780.55	54.52	165.19

## Data Availability

Research data are not shared.

## Supplementary Materials

The following are available online, Figure S1: The formation process of small cage in sI hydrate with CH_4_ guest molecule; Figure S2: The formation process of large cage in sI hydrate with CH_4_ guest molecule; Figure S3: The structural configuration of (a,c) tri-sLs, and (b,d) tri-LsL, shared with three pentagon, and two pentagon and one hexagon water rings, respectively. Table S1: The equilibrium distances of H_2_O–guest molecules during the fusion process of mono-cages to tri-cages with different guest molecules; Table S2: The stabilization energy (E_sta_, kJ/mol), stabilization energy per H_2_O molecule (E_sta-p_, kJ/mol), and capture energy (E_c_, kJ/mol) of capturing guest molecules CH_4_/C_2_H_6_ one by one in double cages structure.
