# Supplementary material for: Theoretical Investigation of the Fusion Process of Mono-Cages to Tri-Cages with CH4/C2H6 Guest Molecules in sI Hydrates
Source: Molecules. 2021 Nov 23;26(23):7071. doi: 10.3390/molecules26237071 (PMC8659103; doi:10.3390/molecules26237071)
Supplement: Supplementary file 1 [file molecules-26-07071-s001.zip › molecules-1457091-supplementary.pdf]

Supporting Information

Theoretical Investigation of the Fusion Process of mono-cages to tri-cages with CH_4/C_2H_6 Guest Molecules in sI Hydrates

Shuxian Wei[a], Siyuan Liu[a*], Shoufu Cao[a], Sainan Zhou[a], Yong Chen[b*], Zhaojie Wang[a], Xiaoqing Lu [a*]

[a]*School of Materials Science and Engineering, China University of Petroleum, Qingdao, Shandong 266580, P. R. China*

[b]*School of geosciences, China University of Petroleum, Qingdao, Shandong 266580, P. R. China*

*Corresponding author: Siyuan Liu, Yong Chen and Xiaoqing Lu

E-mail address: lsy@upc.edu.cn, yongchenzy@upc.edu.cn and luxq@upc.edu.cn

Telephone: 86-532-8698-3372

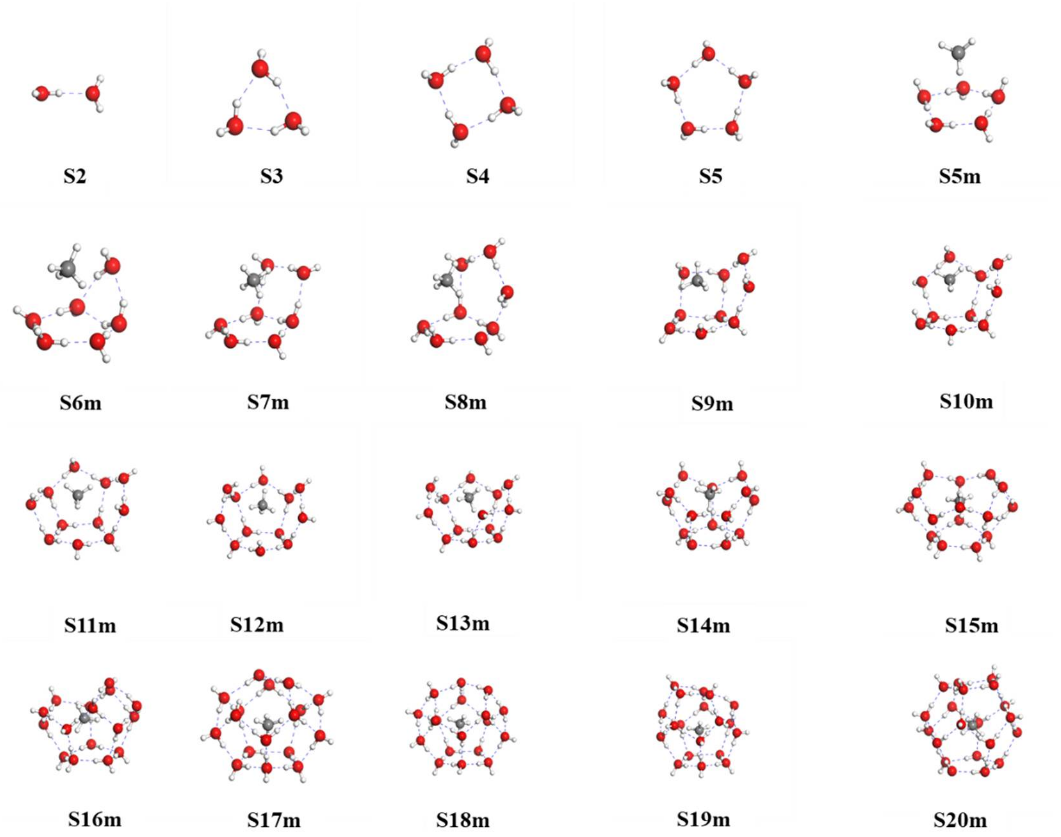

Figure S1. The formation process of small cage in sI hydrate with CH_4 guest molecule (S20m represent small cage, the number of water molecule, and methane guest molecule, respectively).

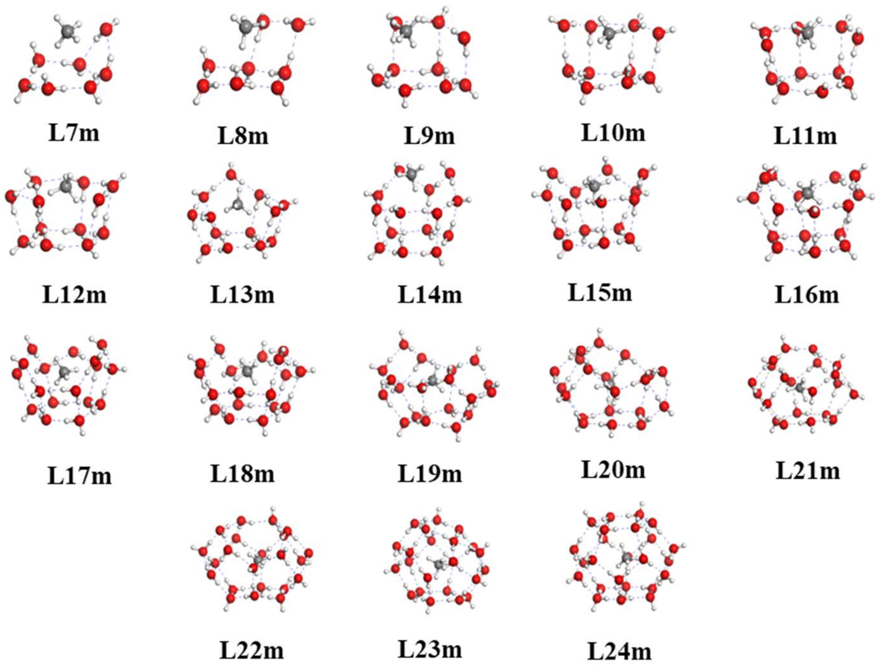

Figure S2. The formation process of large cage in sI hydrate with CH_4 guest molecule (L24m represent large cage, the number of water molecule, and methane guest molecule, respectively).

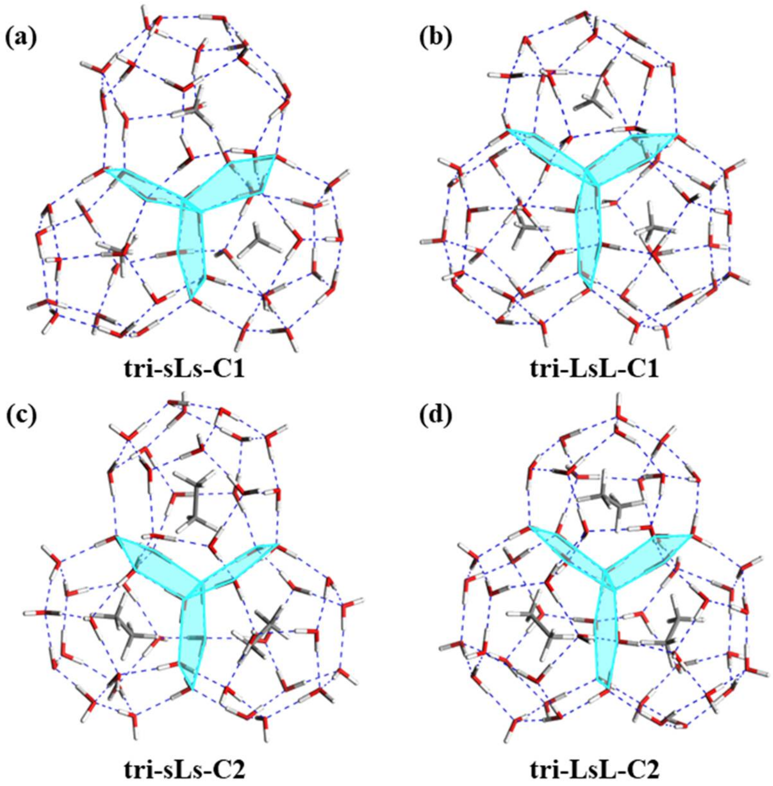

Figure S3. The structural configuration of (a, c) tri-sLs, and (b, d) tri-LsL, shared with three pentagon, and two pentagon and one hexagon water rings, respectively (C1, C2 represent CH_4 and C_2H_6 guest molecule, respectively.).

Table S1. The equilibrium distances of H_2O–guest molecules during the fusion process of mono-cages to tri-cages with different guest molecules. (small-C1 and Large-C1 represent small cage and large cage are occupied by CH_4 guest molecule, and corresponding $d_{C\text{-}O}$ is minimum between carbon atom of CH_4 and oxygen atom of H_2O; small-C2 and Large-C2 represent small cage and large cage are occupied by C_2H_6 guest molecule, and corresponding $d_{C\text{-}O}$ is minimum between centroid of C_2H_6 and oxygen atom of H_2O.)

**Guest molecules**	**Structure**	**$d_{C\text{-}O}$ (Å)**			
		small-C1	small-C2	Large-C1	Large-C2
**CH_4**	mono-cages	3.684	--	3.809	--
	Double-s	3.604	--	--	--
	Double-Ls	3.385	--	3.744	--
	Double-L	--	--	3.744	--
	tri-LsL	3.325	--	3.684	--
	tri-sLs	3.507	--	3.749	--
**C_2H_6**	mono-cages	--	3.652	--	3.805
	Double-s	--	3.662	--	--
	Double-Ls	--	3.752	--	3.822
	Double-L	--	--	--	3.822
	tri-LsL	--	3.540	--	3.680
	tri-sLs	--	3.592	--	3.952

CH_4/C_2H_6	Double-s	3.630	3.706	--	--
	Double-Ls-C1C2	--	3.756	3.724	--
	Double-Ls-C2C1	3.661	--	--	3.783
	Double-L	--	--	3.856	3.908
	tri-LsL-C1C1C2	3.324	--	3.612	3.696
	tri-LsL-C1C2C1	--	3.388	3.665	--
	tri-LsL-C1C2C2	--	3.431	3.631	3.664
	tri-LsL-C2C1C2	3.327	--	--	3.625
	tri-sLs-C1C1C2	3.566	3.563	3.721	--
	tri-sLs-C1C2C1	3.563	--	--	3.966
	tri-sLs-C1C2C2	3.598	3.549	--	3.943
	tri-sLs-C2C1C2	--	3.576	3.717	--

Table S2. The stabilization energy (E_{sta}, kJ/mol), stabilization energy per H_2O molecule ($E_{sta\text{-}p}$, kJ/mol), and capture energy (E_c, kJ/mol) of capturing guest molecules CH_4/C_2H_6 one by one in double cages structure (For example, s-1C1 means the small cage of empty double cages captures CH_4, and sL-C1C2 means the large cage of half-full double cages captures C_2H_6 based on small cage capturing CH_4).

**Guest molecule**	**Structure**		**E_{sta}**	**$E_{sta\text{-}p}$**	**E_c**
**CH_4**	Double-s	s-1C1	1751.68	50.05	-26.98
		s-2C1	1780.12	50.86	-28.43
	Double-Ls	s-1C1	2116.83	54.28	-26.17
		L-1C1	2115.38	54.24	-24.73
		sL-2C1	2141.74	54.92	-24.92
		Ls-2C1	2141.74	54.92	-26.36
	Double-L	L-1C1	2313.97	55.09	-24.22
		L-2C1	2339.38	55.70	-25.41
**C_2H_6**	Double-s	s-1C2	1756.82	50.19	-32.12
		s-2C2	1789.15	51.12	-32.33
	Double-Ls	s-1C2	2115.80	54.25	-25.14
		L-1C2	2126.62	54.53	-35.96
		sL-2C2	2152.63	55.20	-36.84
		Ls-2C2	2152.63	55.20	-26.01
	Double-L	L-1C2	2326.60	55.40	-36.85
		L-2C2	2364.07	56.29	-37.47

	Double-s	s-C1	1751.68	50.05	-26.98
		s-C1C2	1783.62	50.96	-31.94
		s-C2	1756.82	50.19	-32.12
		s-C2C1	1783.62	50.96	-26.80
**CH_4/C_2H_6**	Double-Ls	s-C1	2116.83	54.28	-26.17
		s-C2	2115.80	54.25	-25.14
		L-C1	2115.38	54.24	-24.73
		L-C2	2126.62	54.53	-35.96
		sL-C1C2	2153.12	55.21	-36.29
		sL-C2C1	2140.82	54.89	-25.02
		Ls-C1C2	2140.82	54.89	-25.44
		Ls-C2C1	2153.12	55.21	-26.50
	Double-L	L-C1	2313.97	55.09	-24.22
		L-C1C2	2351.56	55.99	-37.59
		L-C2	2326.60	55.40	-36.85
		L-C2C1	2351.56	55.99	-24.96
